# Genetic Variants of Pregnane X Receptor (PXR) and CYP2B6 Affect the Induction of Bupropion Hydroxylation by Sodium Ferulate

**DOI:** 10.1371/journal.pone.0062489

**Published:** 2013-06-19

**Authors:** Lichen Gao, Yijing He, Jie Tang, Jiye Yin, Zhengyu Huang, Fangqun Liu, Dongsheng Ouyang, Xiaoping Chen, Wei Zhang, Zhaoqian Liu, Honghao Zhou

**Affiliations:** 1 Pharmacogenetics Research Institute, Institute of Clinical Pharmacology, Hunan Key laboratory of Pharmacogenetics, Central South University, Changsha, Hunan, China; 2 Department of Pharmacy, Changsha Central Hospital, Changsha, Hunan, China; 3 Institute for Pharmacogenomics and Individualized Therapy, University of North Carolina at Chapel Hill, Chapel Hill, North Carolina, United States of America; University of Kentucky, United States of America

## Abstract

This study investigated the effects of pregnane X receptor (PXR/*NR1I2*) and *CYP2B6* genetic variants on sodium ferulate (SF)-mediated induction of bupropion hydroxylation. The pharmacokinetics of bupropion and hydroxybupropion were evaluated after an oral dose of bupropion (150 mg) administered with and without SF pretreatment for 14 days in 33 healthy subjects. The area under the time-concentration curve (AUC) ratio of AUC_hyd (AUC_(0-∞)_ of hydroxybupropion)/AUC_bup (AUC_(0-∞)_ of bupropion) represents the *CYP2B6* hydroxylation activity, which was significantly lower in CYP2B6*6 carriers (*NR1I2* TGT noncarriers or carriers) than in noncarriers in both the basal and SF-induced states (p-value<0.05). AUC ratio and AUC_hyd of *NR1I2* -24113AA variant were markedly lower than GA and GG genotypes (7.5±2.1 versus 14.5±3.3 and 20.6±1.1, and 8873±1431 versus 14,504±2218 and 17,586±1046) in the induced states. However, -24020(-)/(-) variant didn't show significant difference in the induction of *CYP2B6* hydroxylation activity by SF compared with other -24020[GAGAAG]/(-) genotypes. *NR1I2* TGT haplotype (-25385T+g.7635G+g.8055T) carriers exhibited a significantly decreased AUC ratio, compared with TGT noncarriers, in the basal states (7.6±1.0 versus 9.7±1.0), while this result wasn't observed in CYP2B6*6 noncarriers. Moreover, individuals with complete mutation-type [CYP2B6*6/*6+*NR1I2* TGT+ -24113AA+ -24020 (-)/(-)] showed even lower percent difference of AUC ratio (8.7±1.2 versus 39.5±8.2) than those with complete wild-type. In conclusion, it is suggested that *NR1I2* variants decrease the bupropion hydroxylation induced by SF treatment, particularly in CYP2B6*6 carriers.

**Trial Registration:**

ChiCTR.org ChiCTR-TRC-11001285

## Introduction

Human pregnane X receptor (hPXR) may regulate metabolic pathways in response to changes in the environment by appropriate alterations in gene expression of key metabolic enzymes [1]. hPXR is a member of the nuclear receptor superfamily, and encoded by the *NR1I2* gene, which is located in the chromosome 13q11-13 and composed of nine exons. It consists of 434 amino acids with a molecular weight of 49.7 kDa [2]. It is abundantly expressed in liver and intestine, and plays an important role in ligand-activated transcription and regulation of genes involved in xenobiotic and endobiotic metabolism [3]–[5]. The transcriptional activity of *NR1I2* is mediated through the ligand binding pathway. Many chemical drugs, including rifampin [4], [5], phenobarbital [6], [7], dexamethasone [7], [8], and herbs such as Kava [7], St. John's wort (hyperforin) [9], [10], and Ginkgo biloba extract (ginkgolide A) [11] have been verified as regulators of *NR1I2* transcriptional activity. Meanwhile, hPXR binds to DNA response elements as a heterodimer with the 9-cis retinoid X receptor (RXRα) [12] and acts as a transcriptional regulator of many important genes that are encoding drug-metabolizing enzymes, such as phase I enzymes (e.g., *CYP3A4*, *3A5*, *2A6*, *2B6*, 2*C9*, *2C19*, and *1A1*), phase II enzymes (e.g., *UGT1A1*, *1A3*, *1A4*, and *1A6*), and phase III enzymes (e.g., *MDR1*, *MRP2*, and *OATP1B1*) [13]–[15].

The genetic variants of *NR1I2* affect the disposition and interaction of various drugs through an induction pathway of CYPs, which may explain the interindividual difference of PXR activity [16], [17]. Previous studies showed that variant alleles of *NR1I2* single nucleotide polymorphisms (SNPs) -25385C>T (rs3814055), -24113G>A (rs2276706), g.7635A>G (rs6785049), or g.8055C>T (rs2276707) were associated with increased *NR1I2* transcriptional activity [18], and -24020[GAGAAG]>(-) (rs3842689) completed loss of *NR1I2* promoter activity in HepG2 cells [19]. *NR1I2* TGT haplotype (-25385T+g.7635G+g.8055T) and -25385C>T variants were also reported to be associated with reduced induction of bupropion hydroxybupropion by rifampin [20]. However, these controversial results of *NR1I2* variants functions *in vitro* and *in vivo* have not been consistently validated.

Sodium ferulate (3-methoxy-4-hydroxy-cinnamate sodium, C_10_H_9_NaO_4_, SF) is the sodium salt of ferulic acid (FA), which is widely distributed in herbs and Chinese formulas such as Ligusticum, Chuanxiong and Chaihu–Sugan–San [21], [22]. It is usually used as food supplements or herbal medicine in countries or areas accepting the theory of Traditional Chinese Medicine (TCM). With the anti-oxidant, anti-atherogenic, anti-platelet clotting, anti-inflammatory, lipid-lowering, cholesterol biosynthesis inhibitory and analgesic effects [23]–[26], FA has the potential to be developed into an effective pure compound for prevention and treatment of cardiovascular diseases. Presently, SF has been approved by State Food and Drugs Administration of China (SFDA) as a clinical therapy for cardiovascular and cerebrovascular diseases [27], [28].

Combination of SF and CYP2B6 substrate drugs has the potential possibility of drug-drug interactions and may lead to undesirable and harmful clinical consequences. Although human CYP2B6 represents approximately 1% of total hepatic CYP content, it shows a relative contribution of 2% to 10% in total hepatic CYP activity, and participates in the metabolism of a variety of substances including bupropion, selegiline, valproic acid, cyclophosphamide, ifosfamide, nevirapine, efavirenz, propofol, ketamine, and synthetic opioid methadone [29]. In particular, the metabolic pathway of hydroxybupropion is almost exclusively catalyzed by CYP2B6 that is a standard model for studies of drug-drug interactions of CYP2B6 substrate drugs [30]–[33]. Recently, we verified that bupropion hydroxylation metabolism (represents CYP2B6 metabolism activity) was induced by a 14 days pre-treatment of 150 mg SF in healthy volunteers [34]. Our previous investigations in HepG2 cells suggested that FA may increase 67% transcriptional expression of *CYP2B6* through PXR activation compared with control group (unpublished data). Due to frequently combinational use, especially in China, of SF and other drugs during the treatment of clinical diseases, it is important to be aware of the possibility of interactions of combination of SF and CYP2B6 substrate drugs, and to prevent harmful clinical toxicity.

Effects of *CYP2B6* variations on protein expression levels and enzyme activities may cause up to hundreds fold of interindividual difference in exposure to drugs [35]. Early studies indicated CYP2B6*6 carriers contributed to the interindividual difference in *CYP2B6* substrate drugs disposition [36], [37]. The low-activity and high-frequency of allele CYP2B6*6 was very prevalent in African Americans (32.8%), Papua New Guineans (62%) and Asians (21%) [35]. It can be simply speculated that the number of abnormal population of *CYP2B6* variants will be marked, when other *CYP2B6* functional variants are added. Therefore, it is very critical to investigate whether *CYP2B6* variants effect the induction of bupropion hydroxylation by SF.

Based on above studies, SF-induced *CYP2B6* activity is suggested to be associated with both CYP2B6*6 polymorphisms and other factors such as *NR1I2* genetic variants. Furthermore, both *NR1I2* and *CYP2B6* variants may be associated with the clinical pharmacokinetics and/or interactions of SF and bupropion. However, clinical pharmacogenetics study data of *NR1I2* and *CYP2B6* variants is still scarce. Further studies are still necessary to investigate the functions of *NR1I2* and *CYP2B6* genetic variants in clinic. The purpose of this paper is to evaluate the effect of *NR1I2* and *CYP2B6* genetic variants, and to demonstrate the relationship between these genetic variants and metabolic induction of bupropion hydroxylation by SF administration in Chinese individuals.

## Materials and Methods

The protocol for this trial and supporting CONSORT checklist are available as supporting information; see Checklist S1 and Protocol S1.

### Genotyping

Genomic DNA was isolated from peripheral blood samples using SQ Blood DNA KitII(Omega Bio-Tic, Georgia, USA).

*NR1I2. NR1I2* -25385C>T (rs3814055), -24113G>A (rs2276706), -24020 [GAGAAG]>(-) (rs3842689), g.7635A>G (rs6785049) and g.8055C>T (rs2276707) were amplified by polymerase chain reaction (PCR) and genotyped. The sequences of forward and reverse primers were 5′-CAAGGCAAGCATCCACTTGA -3′, 5′- GTTGAT- TCTGTTCACTTGGG-3′ for -25385C>T, 5′- AGGCAGCGGCTCCTTGGTAA -3′, 5′-AGGACAGCAGCATGA- CAGTC-3′ for -24113G>A and -24020[GAGAAG]>(-), 5′- CAAGCTCAGTGGGTG- GAGTT -3′, 5′- TTCTCCCCAGGTGAGGATCT-3′ for g.7635A>G and g.8055C>T, respectively. The PCRs used a thermocycling profile of initial denaturation at 95°C for 5 min, followed by 30 cycles of denaturation at 95°C for 30 s, annealing at 55°C for 30 s and extension at 72°C for 1 min. The final volume of the PCR was 50 µl , consisting of 20 ng of DNA, 10 µM of each primer pair, 2.5 µM of dNTPs, 5 µl of 10× reaction buffer, and 2.5 U of Taq DNA polymerase (MBI Fermentas, Ontario, Canada). SNPs were identified by DNA sequencing according to a standard protocol with ABI PRISM Big Dye Terminator Cycle Sequencing Ready Reaction Kit and ABI 3700 DNA Analyzer (Applied Biosystems, Foster City, California, USA).

*CYP2B6*. The wild-type allele CYP2B6*1 was defined as 516G/785A, and CYP2B6*6 was defined as 516T/785G. CYP2B6*6 was detected in a haplotype assay using a two-step allele-specific PCR as described previously [38]. The validity of the method was confirmed by sequencing.

### Subjects

To detect *NR1I2* and *CYP2B6* genetic polymorphisms in the Chinese population, a total of 152 individual samples (from the DNA bank, Hunan Key laboratory of Pharmacogenetics, Central South University) were genotyped. Thirty-four healthy male volunteers (eighteen CYP2B6*1/*1, nine CYP2B6*1/*6 and seven CYP2B6*6/*6) were enrolled in the clinical trial with informed consent form signed (aged 20 to 24 years; weight range: 52–77 kg; body mass index range: 18–25 kg/m^2^), and divided into CYP2B6*6 noncarriers (CYP2B6*1/*1, wild-type) and CYP2B6*6 (CYP2B6*1/*6+CYP2B6*6/*6) carriers. The health status of subjects were ascertained by checking medical history and taking a full clinical examination, drug screening, and standard hematologic and blood chemical laboratory tests. Standardized protein-rich diets with no vegetables, fruits or cereals were provided for subjects for 2 weeks prior to study and during the whole study, in order to exclude the influence of food-originated FA. Drugs, alcohol, soft drinks, tobaccos, vitamins and caffeine-containing beverages, any nutritional supplements were refrained for 2 weeks before study commencement and throughout the study. Regular heavy drinkers, smokers, users of glucocorticoids and those with body weight exceeded their ideal weight by 20% were excluded. Finally, 33 of recruited subjects finished the trial (the date range of subject enrollment was from April 24, 2011 to July 25, 2011).

This study was approved by the ethics committee of Central South University, Changsha, Hunan, P. R. China (approved number: CTXY- 110003) and registered in the Chinese Clinical Trial Registry (registration number: ChiCTR-TRC -11001285, name: The effect of ferulic acid on metabolism of bupropion by *CYP2B6*). Overall clinical trial procedures abided by the Good Clinical Practice of the International Conference on Harmonization (ICH-GCP).

### Study Design

This study was carried out in a two-phase, randomized, crossover manner with a 2-week washout period between phases. In each phase, after an overnight fast, subjects were given pretreatment with or without three 50-mg SF tablets (one tablet, three times a day) of the same batch (Lot No.: 100810; HengDa ShengKang Pharmaceutical Co., Sichuan, China) for fourteen days. The signature of subjects, supervision of investigators and detection of plasma concentrations of FA were carried out to assure subject compliance to treatment. On day 15, after an overnight fast, a single dose of 150 mg bupropion (two tablets of 75 mg Zyban SR; WanTe, Hainan, China) was given to each subject by oral administration with 200 ml water at 8:00 a.m. Subjects fasted for another 4 h after drug administration, except water drinking 2 h after dosing. Standard meals were provided for all of the participants. Serial blood samples for PK analysis (5 ml) were collected using a forearm in-dwelling venous catheter (anticoagulation with sodium heparin) before dosing and at 0.5, 1, 2, 3, 4, 5, 6, 8, 10, 12, 24, 36, 48, 60 and 72 h after bupropion ingestion.

### Drug Concentration Analysis

The plasma concentrations of bupropion and hydroxybupropion were examined by liquid chromatography–mass spectrometry using Waters Micromass Quattro Micro API LC/MS/MS instrument (Milford, MA, USA). An Angel kromasil C18 (5 µm, 150×2.1 mm) and a mobile phase (acetonitrile∶0.1% formic acid∶20 mM ammonium formate = 4∶3∶3) at a flow rate of 0.2 ml/min were applied. Propranolol was used as the internal standard. The ion transitions monitored were as follows: *m/z* 240 to 184 for bupropion,*m/z* 256 to 238 for hydroxybupropion and *m/z* 260 to 183 for propranolol. These transitions represent the product ions of the [M+H]^+^ ions. The lower limits of detection for bupropion and hydroxybupropion were 0.25 ng/ml and 1.168 ng/ml, and the assay ranges used were 0.42–430.1 ng/ml and 2.344–1200 ng/ml, respectively. The linear correlation coefficient for bupropion calibration curves was 0.998, and for hydroxybupropion was 0.996. The highest bupropion and hydroxybupropion plasma concentration measured were 323.70 ng/ml and 615.66 ng/ml. The mean extraction recovery and precision of bupropion and hydroxybupropion was assessed by determining quality control (QC) plasma samples at three concentration levels (concentrations of bupropion and hydroxybupropion were 2.344, 37.5, 600 ng/ml and 0.84, 13.438, 430 ng/ml, respectively). The recovery of bupropion and hydroxybupropion, determined at each concentration level was 92.1±7.1, 93±5.3, 91.8±3.6 and 90.6±4.7, 91.5±4.8, 92.4±6.3 (%, n = 5), respectively, and did not exceed 10% of the relative standard deviation (R.S.D.). The precision (R.S.D.) of bupropion at three concentration levels in intra-day and inter-day was 8.67, 5.67, 7.32 (%) and 4.19, 6.57, 8.92 (%), respectively. Similarly, the precision (R.S.D.) of hydroxybupropion in intra-day and inter-day was 7.34, 3.58, 8.49 (%) and 4.25, 7.13, 9.12 (%), respectively. The precision of both bupropion and hydroxybupropion was less than 10% of R.S.D.

### Pharmacokinetic Analysis

The maximum plasma concentration (*C*_max_) and the time to *C*_max_ (*T*_max_) were obtained by inspection of the concentration-time data. The AUC to the last quantifiable concentration AUC_0-t_ was determined by use of the linear trapezoidal rule. *ke* is the elimination rate constant determined from the terminal slope of the log concentration-time plot. The elimination half-life (*t*_1/2_) was calculated as 0.693/*ke*. The area under the concentration-time curve extrapolated to infinity AUC_0-∞_ was calculated as AUC_0-∞_ = AUC_0–72_+*C*72/*ke*, where *C*72 is the plasma concentration measured 72 h after drug administration. The oral clearance (CL/F) of bupropion was calculated by dividing the bupropion dose by the AUC of bupropion and the subject's weight.

### Statistical Analysis

Study sample sizes were estimated based on prior bupropion PK data. The planned sample size, statistical power, and alpha level were performed using the NCSSV2007 program. For instance, if three subjects in TGT carriers for CYP2B6*6 noncarriers group were enrolled, the power (AUC ratio) is up to 0.92371 (α = 0.05, β = 0.2) according to prior bupropion PK data. Likewise, in TGT carriers for CYP2B6*6 carriers group, if five subjects participated in this study, the power will add to 0.93165 (α = 0.05, β = 0.2) (calculation methods as described NCSS2007 instruction). The bioequivalence approach was used to determine clinically relevant interactions [39]. AUC ratio, namely AUC_hyd (AUC_(0-∞)_ of hydroxybupropion) was divided by AUC_bup (AUC_(0-∞)_ of bupropion) for each period, representing CYP2B6 activity. The percent differences in the PK parameters between the basal and SF-treated states were calculated as an absolute of 100×(induced-basal)/basal. WinNonlin (version 5.2; Pharsight, Mountain View, CA) was used for the PK analysis. The paired two-tailed t-tests were used to determine the difference between basal and induced states, and logarithmic transformation was used for the non–normally distributed data before analysis. The differences in PK parameters between noncarriers and carriers groups of *NR1I2* TGT haplotype and CYP2B6*6 genotypes were obtained using the Wilcoxon rank-sum test. The difference among *NR1I2* -25385C >T (*CC*, *CT* and *TT*) and -24113G>A (*GG*, *GA* and *AA*) genotype groups was obtained by use of the Kruskal-Wallis test. The Fisher's exact test was used to detect difference of genotype distributions between CYP2B6*1/*1 and CYP2B6*1/*6+CYP2B6*6/*6. Results were expressed as mean ± standard deviation in the text and tables, and as mean ± standard error in the figures. Linkage disequilibrium (LD) analysis and haplotype construction were performed using the Haploview 4.2 program (Broad Institute of Harvard and MIT, Cambridge, MA) and Phase 2.0 (UW Center for Commercialization; University of Washington), Data were analyzed with SPSS software (IBM SPSS; version 13.0). The chosen statistical significance level was p<0.05.

## Results

### Genetic Polymorphisms of *NR1I2* in the Chinese Population

The frequencies of *NR1I2* alleles -25385T, -24113A, -24020(-), g.7635G and g.8055T were 0.283, 0.181, 0.208, 0.522, and 0.625, respectively. The distribution of those genotypes was consistent with Hardy-Weinberg equilibrium (p-value >0.05, χ^2^ test). *NR1I2* -25385C>T, -24113G>A, and -24020[GAGAAG]>(-) displayed a slight LD (*r* ^2^≥0.56). Also, *NR1I2* g.7635A>G and g.8055C>T showed a slight LD (*r* ^2^≥0.62). In view that compared with previous *NR1I2* haplotypes investigation, we selected three SNPs -25385C>T, g.7635A>G, and g.8055C>T to perform haplotype analysis, and eight haplotypes were inferred based on these SNPs in Chinese: CAC, CGT, TGT, TAC, CGC, TGC, CAT, and TAT. The population frequencies of these haplotypes were 0.221, 0.491, 0.136, 0.035, 0.073, 0.043, 0.001 and 0.000, respectively. CAC haplotype was regarded as the wild-type allele, and TGT haplotype was the mutation-type allele. The subjects were divided into TGT carrier (n = 10) and noncarrier (n = 23) groups, based on the existence of *NR1I2* TGT haplotype or not.

### Results of the Clinical Study

According to the ICH-GCP guideline [40], no serious drug-related adverse event was observed from the 33 subjects during the course of this study. No clinically significant alterations were observed in heart rate, blood pressure or body temperature. No significant difference was shown in the demographic characteristics of the volunteers or in the distributions of *CYP2B6* genotypes among *NR1I2* haplotypes (see Table 1).

**Table 1:** Demographic data and genotypes of the subjects.

	TGT Noncarriers (n = 23)	TGT Carriers (n = 10)	p
Age (yr)	22±0.6	21±0.3	0.105^a^
Weight (kg)	63±1.7	62±2.3	0.384^a^
High (cm)	1.70±0.01	1.71±0.02	0.603^a^
BMI (kg/m^2^)	22.0±0.4	21.7±0.5	0.124^a^
2B6*1/*1 (18)	14	4	0.448^b^
2B6*1/*6 (9)+*6/*6 (6)	9	6	

a Wilcoxon rank-sum test between TGT Noncarriers and TGT Carriers groups.

b Fisher's exact test between CYP2B6*1/*1 and CYP2B6*1/*6+CYP2B6*6/*6, X^2^ = 1.224, p = 0.448. BMI: body mass index.

After SF treatment, in complete wild-type groups, AUC_bup was significantly lower (657±73 versus 853±102), while AUC_hyd was significantly higher (15,594±2799 versus 12,732±2448). The AUC ratio (AUC_hyd/AUC_bup), which represents the metabolic activity of bupropion into hydroxybupropion, markedly increased (17.7±2.1 versus 13.1±1.7, see Table 2). However, No significant difference was shown in -24113AA, -24020(-)/(-), and TGT carriers (CYP2B6*6 carriers), AUC_hyd and AUC ratio after SF treatment (p-value>0.05, paired t test, see Table 3 and 4). Furthermore, in -24113AA variant, AUC ratio and AUC_hyd was significantly lower (7.5±2.1 versus 14.5±3.3 and 20.6±1.1, and 8873±1431 versus 14,504±2218 and 17,586±1046) than -24113GA and GG genotypes in the induced states. Moreover, -24113 AA variant also showed significantly lower AUC ratio (6.8±0.7 versus 11.3±2.5 and 15.5±0.8) than -24113GA and GG genotypes in the basal states (see Table 3). Further research found that -24020(-)/(-) variant only showed slight difference (8895±1328 versus 15,116±1797 and 15,518±1168, p-value>0.05) in AUC_hyd in the induced states compared to other -24020[GAGAAG]/(-) genotypes, and no significant impact was shown on the induction of bupropion hydroxybupropion by SF compared with other -24020[GAGAAG]/(-) genotypes.

**Table 2:** Effects of complete wild-type and mutation-type individuals on SF-mediated metabolic induction of bupropion hydroxylation.

	*Complete wild-types* (n = 6)	*Complete mutation-types* (n = 2)
AUC _(0–∞)__bup (ng·h/ml)		
Basal	853±102	1262±344^a^
Induced	657±73	1191±254^a^
% difference^b^	26.9±0.7*	5.4±0.6^a^
AU C_(0–∞)__hyd (ng·h/ml)		
Basal	12,732±2448	6523±384^a^
Induced	15,594±2799	7562±890^a^
% difference^b^	25.2±5.9*	15.5±6.8^a^
AUC_hyd/AUC_bup		
Basal	13.1±1.7	7.0±1.4^a^
Induced	17.7±2.1	7.6±1.6^a^
% difference^b^	39.5±8.2*	8.7±1.2^a^

* p<0.01, paired t test between the basal and induced states.

a p<0.05, Wilcoxon rank-sum test between [CYP2B6*1/*1+*NR1I2* CAC + -24113GG+-24020[GAGAAG]/[GAGAAG]] (complete wild-type) and [CYP2B6*6/*6+*NR1I2* TGT+ -24113AA+-24020(-)/(-)] (complete mutation-type) groups.

b % difference represents the percent difference between basal and induced state, calculated as an absolute of 100×(induced-basal)/basal.

**Table 3:** Effects of *NR1I2* SNP polymorphisms on SF-mediated metabolic induction of bupropion hydroxylation.

	-24113GG (n = 23)	-24113GA (n = 7)	-24113AA (n = 3)	-24020[GAGAAG]/[GAGAAG] (n = 22)	-24020[GAGAAG]/(-) (n = 8)	-24020(-)/(-) (n = 3)
AUC_(0–∞)__bup (ng·h/ml)						
Basal	977±64	1062±136	1138±207	1011±64	1114±131	1125±186
Induced	960±55	1031±169	1083±206	995±53	1000±157	1086±214
% difference^b^	8.9±1.5	0.6±0.4	0.5±0.3	6.8±1.3	4.3±0.5	1.2±0.4
AU C_(0–∞)__hyd (ng·h/ml)						
Basal	13,296±881	11,601±1367	8126±1573	12,063±952	11,814±1101	8185±1436
Induced	17,586±1046	14,504±2218	8873±1431^a^	15,518±1168	15,116±1797	8895±1328
% difference^b^	32.0±5.1*	27.9±6.9*	12.5±6.3	31.2±5.3*	28.0±6.0*	17.5±4.7
AUC_hyd/AUC_bup						
Basal	15.5±0.8	11.3±2.5	6.8±0.7^a^	12.4±1.0	11.9±1.9	7.3±0.8
Induced	20.6±1.1	14.5±3.3	7.5±2.1^a^	16.8±1.2	15.6±3,1	9.5±2.0
% difference^b^	34.4±5.0*	30.6±6.3*	11.3±7.9	39.4±5.2*	28.6±4.9*	20.6±8.5

* p<0.01, paired t test between the basal and induced states.

a p<0.05, Kruskal-Wallis test for *NR1I2* -24113G*>*A and -24020[GAGAAG]*>*(-) groups.

b % difference represents the percent difference between basal and induced state, calculated as an absolute of 100×(induced-basal)/basal.

**Table 4:** Effects of *NR1I2* haplotype and *CYP2B6* genotypes on SF -mediated metabolic induction of bupropion hydroxylation.

	*CYP2B6* * *6* noncarriers (wild-type) (n = 18)		*CYP2B6* * *6* carriers (n = 15)		p^d^
	TGT Noncarriers (n = 14)	TGT Carriers (n = 4)	TGT Noncarriers (n = 9)	TGT Carriers (n = 6)	
AUC_(0–∞)__bup (ng·h/ml)					
Basal	869±63	924±73	1186±100^b^	1247±161	**0.004**
Induced	800±99	898±51	1175±81^b^	1194±90	**0.002**
% difference^e^	7.9±0.8	2.8±0.5	2.2±0.8	1.0±0.8	0.229
AU C_(0–∞)__hyd (ng·h/ml)					
Basal	12,484±1239	12,057±1100	11,485±1499	8844±822^c^	**0.044**
Induced	16,759±1624	14,078±676	14,030±1891	11,029±1225^c^	**0.021**
% difference^e^	32.5±6.5*	18.1±5.2^a^	24.9±9.1*	24.4±7.5	**0.048**
AUC_hyd/AUC_bup					
Basal	14.5±1.5	13.9±1.1	9.7±1.0^b^	7.6±1.0^a^^, ^ c	**0.000**
Induced	19.1±1.9	18.6±2.7	11.6±1.1^b^	9.1±1.5^c^	**0.000**
% difference^e^	34.8±7.1*	30.1±6.7*	23.2±7.0	17.1±9.5^a^	**0.012**

* p<0.01, paired t test between the basal and induced states.

a p<0.05, Wilcoxon rank-sum test for the *NR1I2* groups within each *CYP2B6* genotype group.

b p<0.05, Wilcoxon rank-sum test for the *CYP2B6* groups with TGT noncarriers.

c p<0.05, Wilcoxon rank-sum test for the *CYP2B6* groups with TGT carriers.

d Wilcoxon rank-sum test for the *CYP2B6* groups; p<0.05 is indicated in bold.

e % difference represents the percent difference between basal and induced state, calculated as an absolute of 100×(induced-basal)/basal.

The effects of *CYP2B6* genotypes and *NR1I2* TGT haplotype were considered as a whole and are showed by subgroups (see Table 4). CYP2B6*6 carriers always showed significantly lower AUC ratio than that of noncarrier group in both the basal and induced states, and also in TGT noncarrier or carrier groups. *NR1I2* TGT carriers (CYP2B6*6 carriers) had a significantly lower AUC ratio and percent difference of AUC ratio (7.6±1.0 versus 9.7±1.0, and 17.1±9.5 versus 23.2±7.0) than noncarriers. However, there was no significant difference observed in AUC ratio and percent difference of AUC ratio between *NR1I2* TGT carrier and noncarrier groups for CYP2B6*6 noncarriers. Furthermore, TGT carriers (CYP2B6*6 noncarriers) had significantly lower percent difference of AUC_hyd (18.1±5.2 versus 32.5±6.5) than noncarriers, while there was no significant difference in CYP2B6*6 carriers (see Table 4). In addition, the complete mutation-type [CYP2B6*6/*6+*NR1I2* TGT+ -24113AA+-24020(-)/(-)] individuals exhibited even lower percent difference of AUC ratio (8.7±1.2 versus 39.5±8.2) than those of complete wild-types (p-value<0.05, see Table 2).

**Figure 1:**
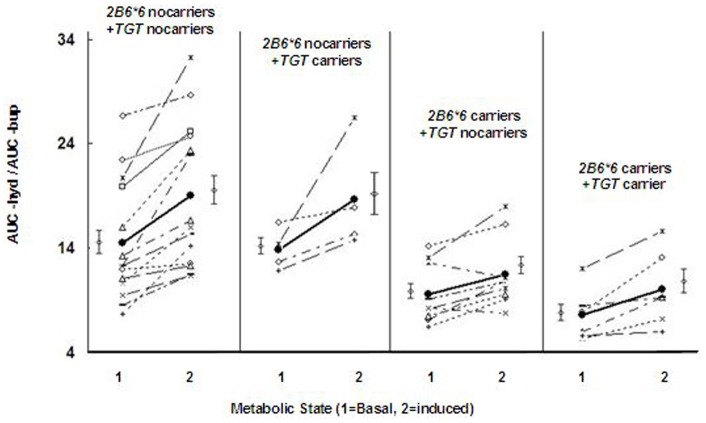
Effects of *NR1I2* TGT and CYP2B6*6 on induction of bupropion hydroxylation by SF. Individual profiles showing AUC_hyd/AUC_bup ratios for the basal and SF-induced states in the *NR1I2* and *CYP2B6* genotype groups (n = 33).

**Figure 2:**
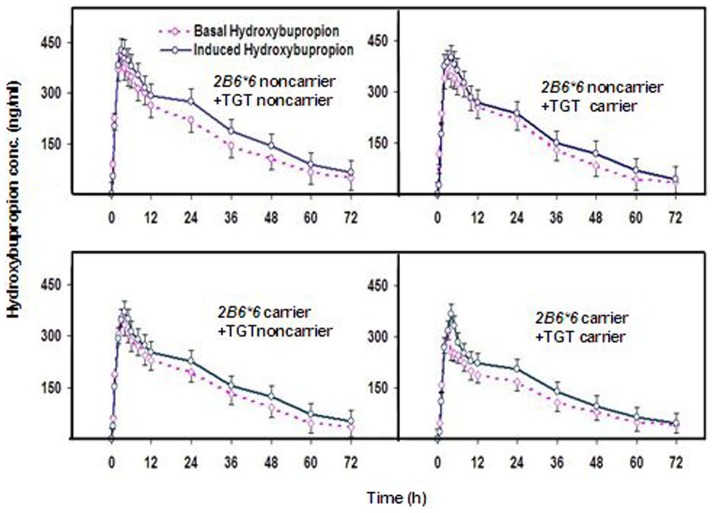
Concentration (conc.)–time profiles of hydroxybupropion. Mean plasma concentration-time profiles of hydroxybupropion after oral administration of 150 mg of bupropion or bupropion+SF treatment in subjects after 14 days in the *NR1I2* and *CYP2B6* genotype groups.

As shown in Fig. 1, individual plots of *CYP2B6* and *NR1I2* variants indicated that the combination of *NR1I2* TGT haplotype and CYP2B6*6 affected the AUC ratio in both the basal and induced states. The concentration-time profiles of hydroxybupropion were very different for CYP2B6*6+*NR1I2* TGT carriers from other groups, with the lowest values in both the basal and induced states (see Fig. 2). The Cmax values of hydroxybupropion in the basal and induced states showed no significant difference between *NR1I2* TGT carriers and noncarriers (unpublished data). However, the hydroxybupropion Cmax in CYP2B6*6+*NR1I2* TGT carriers was significantly lower than CYP2B6*6+*NR1I2* TGT noncarriers in both the basal and induced states (284.3±40.8 versus 395.2±40.5, and 332.9±27.6 versus 424.5±32.8, p-value<0.05).

## Discussion

In the present study, we investigated the *NR1I2* genetic polymorphism in Chinese population and found that the allelic frequencies of -25385T and -24113A in Chinese (0.283, 0.181) were lower than the reported frequencies of Korean (0.32, 0.32), European descent (0.39, 0.32), and Africa American (0.39, 0.32) [18], [20]. The allelic frequency of -24020(-) was significantly lower (0.218) than that of Japanese (0.274) and Korean (0.319) [19], [20], and the allelic frequency of g.7635G was an intermediate value (0.522) between European descent (0.35) and Africa American (0.77). However, g.8055T has markedly higher (0.625) allele frequency compared with that in Brazilian (0.125), European descent (0.15), African (0.18), Indian (0.24), Korean (0.41), and Malay (0.43) [18], [20], [41], [42]. The *NR1I2* allelic frequency observed in this study was very similar to the previous report in Chinese [43].

Further haplotypes analysis showed that compared with the Korean, the composition and frequencies of *NR1I2* TGT haplotypes in Chinese people were completely different. Eight haplotypes were inferred based on SNPs in positions -25385C>T, g.7635A>G, and g.8055, and the frequency distribution of TGT haplotype was slightly lower (0.136) than that of Korean (0.199). Furthermore, linkage manners of *NR1I2* SNPs were inconsistent with the Korean. A slight but not complete LD among -25385C>T, -24113G>A, and -24020[GAGAAG]>(-) and between g.7635A>G and g.8055C>T was observed [20]. These results suggest that the *NR1I2* gene has unique characteristics of high polymorphism and significant interethnic variants.

As shown in Table 4, we found in the clinical investigation that in TGT noncarriers (TGT noncarriers, n = 14), the overall pharmacokinetic parameters of bupropion and hydroxybupropion (AUC_bup, AUC_hyd, and AUC ratio) indicated the strongest effects including basal activities, induced activities and their percent differences. However, with the emergence of TGT and CYP2B6*6 variants (TGT carriers, n = 4 and TGT noncarriers, n = 9, respectively), the strongest effects of the overall pharmacokinetic parameters of bupropion and hydroxybupropion became weaker and smaller, until TGT variant existed in CYP2B6*6 carriers (TGT carriers, n = 6). Moreover, in each step of the attenuation effects of the pharmacokinetic parameters, TGT and CYP2B6*6 carriers always showed smaller effects than noncarriers. Therefore, this result suggests that the decreased metabolism of bupropion with SF treatment is affected by both *NR1I2* TGT and CYP2B6*6 variants. Also, similar findings were obtained from -24113G>A, -24020 [GAGAAG]> (-), and complete wild/mutation-type individuals (see Table 2 and 3). In short, our data strongly support the hypothesis that *NR1I2* TGT haplotype, -24113AA, and CYP2B6*6 variants play very important roles in bupropion disposition.

Interestingly, in previous study, *NR1I2* TGT carriers slightly manifested stronger effects on some pharmacokinetics parameters of bupropion and hydroxybupropion than the corresponding noncarrier groups (p-value>0.05). Conversely, CYP2B6*6 carriers showed smaller effects on AUC_hyd, and AUC ratio than the noncarriers (p-value<0.05) [20]. Reports also showed that CYP2B6 expression increased in the basal state while decreased in the induced state when treated with rifampin in PXR.2 cells [44]. However, these results were not observed in our study. Rifampin is a known selective human PXR activator with little cross-interaction with other receptors, such as small heterodimer partner (SHP) and hepatocyte nuclear factor-4α(HNF-4α) [45], [46]. Early study indicated that interaction of PXR with HNF-4αand its coactivators, peroxisome proliferator-activated receptor-γ-coactivator-1α(PGC-1α) contributed to the strong induction of CYP3A4 by rifampin, whereas gene expression of SHP was simultaneously inhibited by PXR, which weakened inhibitory effect of SHP on CYP3A4 expression and strengthened the HNF-4αinducibility of CYP3A4 [45], [47]. Therefore, we may assume that under the situation that some interfering factors of SHP gene expression exceeded the effects of PXR variants (PXR function variant led to increased SHP gene expression), the activity of CYP did not decline but increased. More attention should be paid to the study of SHP gene expression and regulation in the near future. Moreover, Owen's group reported that genetic variability in constitutive androstane receptor (*CAR*) was involved in the metabolism and disposition of *CYP2B6* substrate drugs recently [48].

In our study, further analysis showed that TGT carriers, only in the basal states, had significantly lower AUC ratio and percent differences (7.6±1.0 versus 9.7±1.0, and 17.1±9.5 versus 23.2±7.0) than TGT noncarriers. However, CYP2B6*6 carriers exhibited significant differences in the most of pharmacokinetic parameter values of bupropion and hydroxybupropion (AUC_bup, AUC_hyd, and AUC ratio) compared with CYP2B6*6 noncarriers in both the basal and induced states (see Table 4). In addition, from Fig. 1 and Fig. 2, a tenuous distinction existed in the concentration (conc.) – time curves of hydroxybupropion and AUC ratio when TGT carriers appeared; while the curves and AUC ratio values quickly dropped when CYP2B6*6 carriers came. This result suggests that CYP2B6*6 variants had stronger reduced metabolic capacity than *NR1I2* TGT haplotype. Interestingly, the complete mutation-type [CYP2B6*6/*6+*NR1I2* TGT+ -24113AA+-24020(-)/(-)] individuals indicated even lower metabolism activities (8.7±1.2 versus 39.5±8.2) than the complete wild-types (see Table 2). Therefore, we tentatively conclude that reduced metabolic capacity is more significant in individuals including CYP2B6*6 mutations, *NR1I2* TGT haplotype, and other *NR1I2* variants with reduced functions.

To date, the verified sites and positions of the SNPs (or haplotypes) of *NR1I2* functional variants were as follows: -25385C>T (5′-UTR), -24622A>T (5′-UTR), -24446C>A (Exon 1), -24113G>A (Intron 1), -24020[GAGAAG]>(-) (Intron 1), 106G>A (*2, Exon 3), 2904C>T (*5, Exon 3), 4321G>A (*4, Exon 4), 4374G>A (*10, Exon 4), 4444A>G (*11, Exon 4), 7635A>G (Intron 5), 8055C>T (Intron 6), 8528G>A (*12, Exon 8), 8561C>T (*7, Exon 8), 9863A>G (*8, Exon 9), 10620C>T (3′-UTR), 10799G>A (3′-UTR), 11156A>C (3′-UTR), 11193T>C (3′-UTR), and NR1I2*1B (8055C>T, Intron 6+2654T>C, 3′-UTR) [18], [20], [42], [43], [49], [50]. However, some investigations of clinical pharmacogenetics of the *NR1I2* functional variants were not consistent with their findings *in vitro*. For instance, *NR1I2* -25385C>T, -24113G>A, 7635A>G, or 8055C>T was reported to be associated with higher magnitude of induction of intestinal CYP3A by rifampin *in vitro* [18], but recently, the subjects with -25385C>T or TGT (-25385T+g.7635G+g.8055T) carriers were verified to have decreased *CYP2B6* activity (AUC ratio) induced by rifampin in Korean, and NR1I2*1B (8055C>T+2654T>C) haplotype was strongly associated with its downstream target genes of *MDR1* in Asian breast cancer patients [42]. Moreover, the result for our subjects with -24113AA showed the lowest percent differences of AUC ratio (11.3±7.9) after SF induction compared with wild genotypes (see Table 3). The specific mechanism of this difference *in vitro* and *in vivo* is yet unknown. However, we must admit that the result *in vitro* was easily interfered by diverse uncontrollable factors. These results highlighted the important role of *NR1I2* pharmacogenetics in the disposition of putative drug substrates. It can be assumed that *NR1I2* genetic polymorphisms will play an essential role in affecting interethnic variations in drug disposition.

Previous researches reported that oxysterol, 24(S), 25- epoxycholesterol (LXR agonists), glucocorticoid (GR agonists), and vitamin D (VDR agonists) could induce expression of *CYP2B6* through the binding co-activators of the corresponding ligands and PXR [51]–[53]. Moreover, gender factor also affected the results of clinical trials [54], [55]. It is worth mentioning that these interference factors were well balanced through our strict subject exclusion criteria and good clinical trial control. However, there are also some deficiencies in our study. For instance, we paid more attention to the *NR1I2* variants, which had the functions reported *in vitro* or *in vivo* such as -25385C>T, -24113G>A, -24020[GAGAAG]/(-), 7635A>G, and 8055C>T [18], [19], [20], [42], [49], [50], [56]–[58]. The rarely reported or less concerned *NR1I2* variants were not included in this research. Future clinical pharmacogenetics research of *NR1I2* variants should be focused on the reported functional variants with lower distribution frequencies, which have potential possibility to play more important roles than the star variants. In addition, lower concomitance mutation frequencies and strict subject exclusion criteria also affected our subjects enrollment. Relatively small and uneven numbers of individuals with various genotypes were investigated in our study, which were the limitations of drawing of conclusions based upon this sample sizes. Further clinical studies of the *NR1I2* variants pharmacogenetics should be operated in larger groups, and even different ethnic populations [59].

## Conclusions

As the data indicated, *NR1I2* TGT haplotype, -24113AA, CYP2B6*6, and the complete mutation-type [CYP2B6*6/*6+*NR1I2* TGT+ -24113AA+-24020 (-)/(-)] individuals have strong evidence to show the ability to reduce the metabolic capacity of *CYP2B6* after SF administration in Chinese individuals. Individuals/ethnic populations with different genetic backgrounds may show significant differences in drug metabolism and efficacy, sometimes even manifested as severe adverse drug reactions or no efficacy. Our findings provide an important reference for carrying out the gene oriented individual/interethnic therapy of *CYP2B6* substrate drugs, and avoiding the adverse effects of SF and *CYP2B6* substrate drugs combination. Whether other *NR1I2* and regulator variants also have impact on the disposition of *CYP2B6* substrate drugs by SF requires further exploration. Large-scale population pharmacokinetic, pharmacodynamic, and nosazontology analysis using pharmacogenomics method are needed to clarify the role of *NR1I2* and CYP2B6*6 variants in the efficacy, safety, and drug interactions of *CYP2B6* substrate drugs, even disease susceptibility between individuals.

## Supporting Information

Checklist S1 **CONSORT checklist.**
(DOC)

Protocol S1 **Trial protocol.**
(DOC)
