# Supplementary material for: Genetic Variants of Pregnane X Receptor (PXR) and CYP2B6 Affect the Induction of Bupropion Hydroxylation by Sodium Ferulate
Source: PLoS One. 2013 Jun 19;8(6):e62489. doi: 10.1371/journal.pone.0062489 (PMC3686783; doi:10.1371/journal.pone.0062489)
Supplement: Protocol S1 — Trial protocol. (DOC) [file pone.0062489.s002.doc]

**Trial Protocol**

**Allocation**

**Analysis**

**Follow-Up**

**Enrollment**

Assessed for eligibility (n=34)

Excluded (n=1)

♦  Not meeting inclusion criteria (n= 1 )

♦  Declined to participate (n= 0 )

♦  Other reasons (n= 0 )

Analysed (n=33)
♦ Excluded from analysis (give reasons) (n=0)

Lost to follow-up (give reasons) (n=0)

Discontinued intervention (give reasons) (n=0)

Allocated to intervention (n=33)

♦ Received allocated intervention (n=33)

♦ Did not receive allocated intervention (give reasons) (n= 0)

Lost to follow-up (give reasons) (n=0)

Discontinued intervention (give reasons) (n=0)

Allocated to intervention (n=33)

♦ Received allocated intervention (n=33)

♦ Did not receive allocated intervention (give reasons) (n=0)

Analysed (n=33)
♦ Excluded from analysis (give reasons) (n=0)

Randomized (n=33)
